# Multi-Parental Populations Suitable for Identifying Sources of Resistance to Powdery Mildew in Winter Wheat

**DOI:** 10.3389/fpls.2020.570863

**Published:** 2021-01-21

**Authors:** Nana Vagndorf Nordestgaard, Tine Thach, Pernille Sarup, Julian Rodriguez-Algaba, Jeppe Reitan Andersen, Mogens Støvring Hovmøller, Ahmed Jahoor, Lise Nistrup Jørgensen, Jihad Orabi

**Affiliations:** ^1^Nordic Seed A/S, Odder, Denmark; ^2^Department of Agroecology, Aarhus University, Slagelse, Denmark; ^3^Department of Plant Breeding, The Swedish University of Agricultural Sciences, Alnarp, Sweden

**Keywords:** GWAS, Fungal disease, powdery mildew, wheat, multi-parental population

## Abstract

Wheat (*Triticum aestivum* L.) is one of the world’s staple food crops and one of the most devastating foliar diseases attacking wheat is powdery mildew (PM). In Denmark only a few specific fungicides are available for controlling PM and the use of resistant cultivars is often recommended. In this study, two Chinese wheat landraces and two synthetic hexaploid wheat lines were used as donors for creating four multi-parental populations with a total of 717 individual lines to identify new PM resistance genetic variants. These lines and the nine parental lines (including the elite cultivars used to create the populations) were genotyped using a 20 K Illumina SNP chip, which resulted in 8,902 segregating single nucleotide polymorphisms for assessment of the population structure and whole genome association study. The largest genetic difference among the lines was between the donors and the elite cultivars, the second largest genetic difference was between the different donors; a difference that was also reflected in differences between the four multi-parental populations. The 726 lines were phenotyped for PM resistance in 2017 and 2018. A high PM disease pressure was observed in both seasons, with severities ranging from 0 to >50%. Whole genome association studies for genetic variation in PM resistance in the populations revealed significant markers mapped to either chromosome 2A, B, or D in each of the four populations. However, linkage disequilibrium between these putative quantitative trait loci (QTL) were all above 0.80, probably representing a single QTL. A combined analysis of all the populations confirmed this result and the most associated marker explained 42% of the variation in PM resistance. This study gives both knowledge about the resistance as well as molecular tools and plant material that can be utilised in marker-assisted selection. Additionally, the four populations produced in this study are highly suitable for association studies of other traits than PM resistance.

## Introduction

Wheat (*Triticum aestivum* L.) is one of the world’s staple food crops and is responsible for feeding nearly 35% of the population (Paux et al., 2008). One of the most devastating foliar diseases attacking wheat is powdery mildew (PM) caused by the fungal pathogen *Blumeria graminis* f.sp. *tritici*. Infected wheat crops have reduced grain quality and yield losses of up to 34% have been reported (Alam et al., 2011). Symptoms of PM are white powder-like colonies on leaves and stem which consists of mycelium and conidia. These symptoms may be followed by black overwintering sexual structures, cleistothecia, which may overwinter, release ascospores, and infect new host plants in the following growing season. The life cycle of PM contains two important steps; infection and reproduction (Glawe, 2008). The infection step is initiated by an ascospore or conidium landing on a susceptible host, followed by germination and penetration of the epidermal cells of the leaf. Several hyphae elongate and form colonies on the surface of a susceptible host, which may result in repeated cycles of infection and conidia multiplication. Additional infections may spread to neighboring plants by conidia being dispersed by wind and rain. The disease is favored by high humidity (>70%) and windy, cool weather (Thomas et al., 2018), often progressing from the lower to the upper leaves. Today’s cultivation of modern crops includes the use of semi-dwarf and dense cultivars in combination with a high nitrogen supply. This practice favors a rapid development of PM and can lead to severe epidemics. Therefore, PM infection of wheat has become a serious problem in modern agriculture. In order to control the disease, application of foliar fungicides is often recommended, but yield responses are variable depending on locality, timing and level of host resistance (Jørgensen et al., 2018). In Denmark, for instance, only a few fungicides are available for controlling PM and (Jørgensen et al., 2018) and use of resistant cultivars is therefore recommended to promote integrated pest management (IPM) practices, which is considered to be the most environmental-friendly and efficient approach to control PM (Bennett, 1984; Uloth et al., 2016).

Host resistance to PM may include race-specific and non-race specific resistance genes. To date, 58 resistance genes (*Pm1*-*Pm58*) conferring resistance to PM in wheat have been identified and mapped (McIntosh et al., 2017). Several of these have been detected in Scandinavian wheat cultivars in the past, either singly or in combinations of multiple genes (Hovmøller, 1989). Additionally, several genes with minor effect have been found (Keller et al., 1999; Chen et al., 2009; Lillemo et al., 2012). These resistance genes are mostly quantitatively inherited and often provide sufficient levels of disease resistance for a longer time compared to race-specific resistance (Leath and Murphy, 1985; Limpert et al., 1987). Only three PM resistance genes, which are identified and mapped, are providing quantitative PM resistance, namely *Pm38*, *Pm39*, and *Pm46* (William et al., 2003; Spielmeyer et al., 2005; Herrera-Foessel et al., 2011). Additionally, these genes show pleiotropic effects on resistance to yellow/stripe rust, brown/leaf rust and black/stem rust. Several major genes have been incorporated in breeding lines using molecular markers (Yi et al., 2008; Wu et al., 2018). However, evolution of pathogen races with new virulence may cause a “breakdown” of the resistance, thereby rendering the wheat lines susceptible to PM (Liu et al., 2015). Thus, it is important to continually search for new resistance genes against PM and to develop molecular tools for fast and efficient introgression of these genes into the breeding lines.

In today’s plant breeding, the limit is no longer the availability of genetic marker information, but rather the availability of adapted genetic material and high-quality phenotyping to establish a marker-trait correlation between genotype and phenotype in breeding populations. An increased focus has been on performing association mapping on multi-parental mapping populations. Compared to bi-parental mapping populations, multi-parental populations have an increased allelic variation allowing higher mapping resolutions. Resistance to PM have mostly been identified and mapped in studies using bi-parental populations and/or association mapping populations (Huang and Röder, 2004; Mater et al., 2004; Miranda et al., 2006; Hua et al., 2009). A limited number of studies in wheat have used a nested association mapping approach in which the populations share a common founder (Bajgain et al., 2016; Li et al., 2016). However, no other studies have used a multi-parental mapping population to identify and map new resistance genes to PM in wheat.

In this study, two Chinese wheat landraces and two synthetic hexaploid wheat lines were used as donors for creating four multi-parental populations. Landraces are often less susceptible to biotic and abiotic stresses compared to modern cultivars (Newton et al., 2010). Moreover, synthetic wheat lines have been verified to contain new sources of resistance to both biotic and abiotic stresses (Li et al., 2018).

The objectives of this study were to (1) develop four multi-parental populations by crossing Chinese wheat landraces and synthetic hexaploid wheat lines into elite donor lines from Danish plant breeding material, (2) analyze population structure of the multi-parental populations, (3) assess the PM phenotype of individual lines in the multi-parental populations when exposed to natural PM populations under field conditions conducive for PM development, and (4) perform genome-wide association analyses to identify new efficient PM resistance genes.

## Materials and Methods

### Plant Material

**FIGURE 1:**
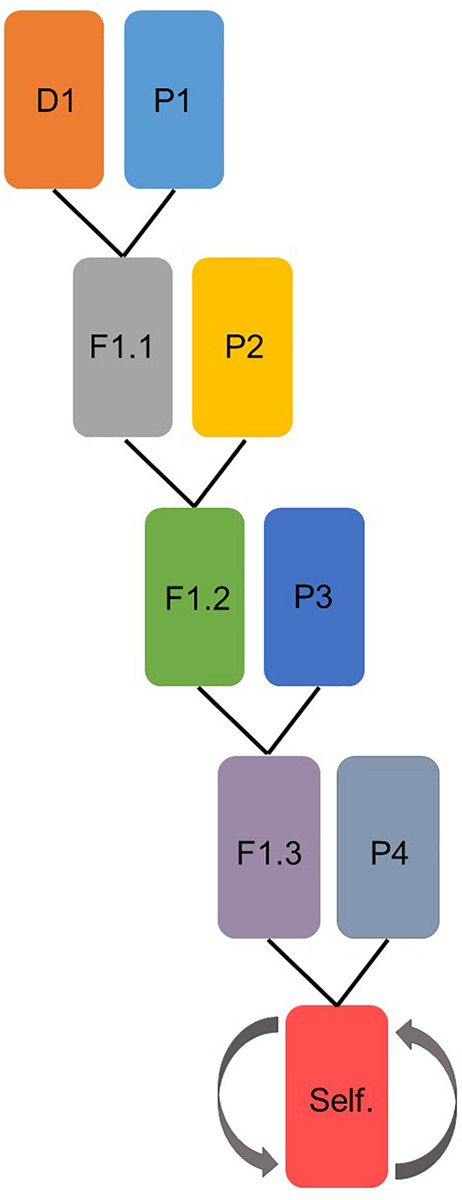
Schematic representation of how each of the four multi-parental populations were prepared. D represents donor line and P the parental cultivars. In the last step, the lines were selfed four times.

Four multi-parental populations were developed by using four different donor lines; two Chinese wheat landraces and two synthetic hexaploid wheat lines (Table 1). The synthetic lines were received from the CIMMYT gene bank and the Chinese landraces were received from the USDA-ARS National Plant Germplasm System (NPGS). The donor lines were tested under field conditions for several diseases e.g., yellow rust, fusarium head blight, tan spot and PM. Donors were chosen based on knowledge concerning desirable resistance traits for several different diseases, aiming to create multi-parent, multi-disease resistant wheat populations (Orabi et al., 2013; Supplementary Table 1). Four elite parental cultivars were used for developing the multi-parental lines in each population (Table 1). After crossing of the last parental cultivar, the multi-parental populations were selfed four times to obtain an acceptable level of homozygosity. In total, 717 lines were produced (Figure 1).

**TABLE 1:** Overview of the pedigree of the four different multi-parental populations.

**Population**	**Pedigree**	**Number of lines**
1	{[(**Chinese Landrace I** *Torp)*NOS 14095.23]*Capricorn}*Sheriff	184
2	{[(**Synthetic I** *Nakskov)*Capricorn]*Torp}*Sheriff	181
3	{[(**Synthetic II** *Torp)*Torp]*Capricorn}*Sheriff	178
4	{[(**Chinese Landrace II** *Nakskov)*Torp]*Capricorn}*Sheriff	174
Total		717

*Donor lines are indicated in bold.*

### Field Trials

Field trials were carried out in two consecutive seasons in 2017 and 2018 in sandy loam at Jyndevad (lat. 54.9°, long. 9.13°), Denmark, an Aarhus University field trial site. This trial site was specifically chosen because it is known to be highly conducive to PM epidemics. Irrigation was setup in the sandy loam during the cropping season when needed.

Multi-parental populations were randomized individually and tested in two replications per year, sown out in September 2016 and October 2017, respectively. Each line was sown in one row as a small plot in 1 m^2^ plots containing six individual lines. Disease reference plots consisting of wheat cultivar “Sheriff” (PM resistant cultivar) and “Mariboss” (PM susceptible cultivar) were placed randomly in the field trial. In the growing season 2018, fungicides were applied between the first and the second PM assessment in order to control two other plant diseases, i.e., septoria tritici blotch and yellow rust.

In total, 726 lines were PM phenotyped, including 717 lines from the multi-parental populations and nine donors and parents (Table 1). Each line was assessed by scoring PM severity using a % scale of diseased leaf area (corresponding 1–9 UPOV scale shown in hard brackets,^1^): 0 = no colonies, 0.01 = 1 or very few colonies [1], 0.1 = few colonies per plant [2], 0.5 = few colonies per tiller [3], 1 = several colonies per tiller [4], 5 = lower leaves up to 10–25% coverage [5], 10 = lower leaves 25% coverage or more [6], 25 = lower leaves 50% coverage or more [7], 50 = half of the leaf area diseased [8], 75 = almost no green leaf area left and 100 = total senescence [9]. Mid-point % values between two adjacent steps were applied when appropriate. Assessments were repeated twice and initiated depending on the development of PM on the susceptible reference plots. The approximate growth stages (GS) at assessment dates in 2017 were GS 37–38 and GS 57–61, respectively, and in 2018 GS 39–49 and GS 49–59, respectively. PM phenotypes are available in a tab separated text file in Supplementary Table 1.

### Genotyping

DNA was purified from the 726 lines using a modified cetyl trimethyl ammonium bromide (CTAB) procedure (Saghai-Maroof et al., 1984) and sent to TraitGenetics (Gatersleben, Germany) for genotyping by using 15 + 5 K Infinium iSelect HD Custom Genotyping BeadChip SNP. In total, 1,7267 molecular markers were included on the array. Individuals missing more that 20% of the data were excluded. Additionally, for the analysis on individual populations markers with a minor allele frequency less than 5% were not included, for the analysis of all populations combined the minor allele frequency was set to 2%. Lastly, unmapped markers were excluded from the genome-wide association analysis.

### Population Structure

A principal component analysis (PCA) was performed on the genotypic data (VanRaden, 2008) and the first two components were plotted against each other. The principal coordinates were calculated on the centered and scaled genotype matrix and plots were generated using R (v. 3.5.0).

### Genomic Heritability

Using the R package “qgg” (Rohde et al., 2020), we fitted a GBLUP for each year and scoring, to estimate narrow sense heritability on the line level.

*Y* = μ + *g* + *c* + ε (M1)

where μ was the general mean, *g* was the genomic line effect, *c* was the blocks and ε was the residuals. All effects in the model were set as random effects. The random effects and residuals were assumed to be independent normally distributed variables described as follows: g ∼ N (0, G σ^2^*_*G*_*), *c* ∼ N (0, I σ^2^*_*r*_*), and *e* ∼ N (0, I σ^2^*_*e*_*). G was the genomic relationship matrix calculated using principles described by VanRaden (2008). Narrow sense genomic heritabilites for the line level were calculated as $(\hat{{\text{h}}^{2}}=\hat{\sigma_{u}^{2}}\bar{d\,\left(\text{G}\right)}/\left(\hat{\sigma_{u}^{2}}\bar{d\,\left(\text{G}\right)}+\frac{\hat{\sigma_{c}^{2}}}{n_{c}}+\frac{\hat{\sigma_{e}^{2}}}{n_{e}}\right)$ where *n*_*c*_ and *n*_*e*_ was the average number of observations per line for block and residuals, respectively, $\bar{d\,\left(\text{G}\right)}$ was the average of diagonal elements in G.

### Genome-Wide Association Analysis

In order to combine the assessments, replicates and year effects on the disease phenotyping, the effect of genotype and environment were corrected by fitting the data to a linear mixed model using R (v. 3.5.0):

*Y* = μ + *Ye* + *G* + *YeRS* + *YeG* + ε (M2)

where μ was the general mean, *Ye* was the years (2017 and 2018), *G* was the genotypes, *R* was the replications, *S* was the scoring date and ε was the residuals. *Ye* were set as a fixed effect and *G*, *YeRS*, and *YeG* were set as random effects. The random effects and residuals were assumed to be independent normally distributed variables described as follows: *G* ∼ N (0, I σ^2^*_*G*_*), *YeRS* ∼ N (0, I σ^2^*_*YeRS*_*), *Y0eG* ∼ N (0, I σ^2^*_*YeG*_*) and *e* ∼ N (0, I σ^2^*_*e*_*).

The corrected values obtained from the model were denoted estimated disease values (EDVs) and were used for GWAS in combination with the genotypic data.

In order to avoid false associations due to population structure, a mixed linear model (MLM) with 1,000 permutations, which includes a kinship matrix, was used in the R package GAPIT (Zhang et al., 2010). Associations with a LOD (logarithm of odds) score above 5.3 were accepted as significant calculated according to the Bonferroni correction for multiple comparisons (Johnson et al., 2010). Physical position of the markers as provided by TraitGenetics (Gatersleben, Germany) was used as mapping information. For the markers with a significant association to PM we checked and updated the mapping information using BLAST. Manhattan plots were generated using the R package “manhattanly” (Bhatnagar, 2016). All markers found to be significant in the GWAS were further analyzed by an ANOVA in R. Explained phenotypic variance was calculated for single and multiple markers to estimate the contribution of each marker and marker combination to the trait (Von Korff et al., 2005). The Linkage disequilibrium (LD) between the best marker representing a quantitative trait loci (QTL) was calculated using the R package “snpStat.”

In addition, to investigate if unequal variances among scoring dates influenced the final results a weighted BLUP model approach was applied as an alternative method to calculate EDVs. Here the Y were weighted according to the residual variance. First, the residual variance within each scoring date was estimated using the model described above (M1). Second, observations were weighted by the ratio between the residual variance of the respective scoring date and the residual variance of the first scoring date, thus putting all observations on the same scale.

$${\text{Y}}_{S}^{w}=Y_{S}*\frac{\sigma_{e\,1}^{2}}{\sigma_{e\,S}^{2}}$$

The resulting weighted observations were then analyzed using a weighted BLUP model in DMU (Madsen and Jensen, 2013).

$$Y_{S}^{w}=\mu +Y\,e+G+Y\,e\,R\,S+Y\,e\,G+\varepsilon /\frac{\sigma_{e\,1}^{2}}{\sigma_{e\,S}^{2}}$$

The estimated values for G obtained from this model were used as an alternative EDVs for GWAS in combination with the genotypic data.

## Results

### Powdery Mildew Assessments

The multi-parental populations were tested in field trials in 2017 and 2018. The first season was generally cool and wet, whereas the second growing season was warmer and drier than usual. A high PM disease pressure was observed in both seasons, with severities ranging from 0 to >50% across all 726 lines, and some lines being more resistant or susceptible than the parental and the donor lines. Line narrow sense heritability was 0.82, 0.92, 0.92, and 0.96 for the first scoring in 2017, the second scoring in 2017, the first scoring in 2018, and the second scoring in 2018, respectively.

**FIGURE 2:**
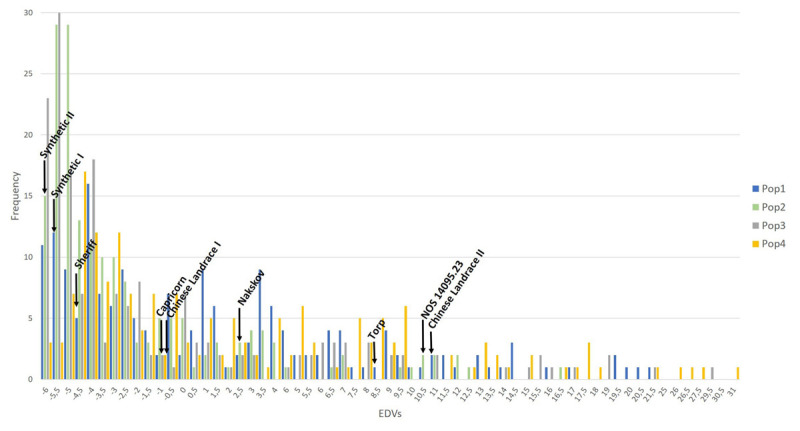
The frequency distribution of the EDVs calculated based on the powdery mildew severity assessed in 2017 and 2018. The populations are color-coded. The EDV from the parent and the donor lines used for generating the multi-parental NAM populations are indicated on the graph.

The EDVs were plotted population-wise in order to reveal the frequency distribution of the disease assessments (Figure 2). Additionally, the EDV of each donor and parental cultivar was indicated in the plot for comparison to the individual multi-parental lines within the four multi-parental populations.

The frequency distribution of the EDVs was shifted toward the lower values on the axis (Figure 2). Population 2 and 3 with the synthetic wheat lines as donors resulted in the highest number of resistant lines. The EDV distribution ranged from −6 to 31 and reflects the deviation from the disease mean of the populations rather than the percentage of disease on each line. However, a normal distribution was detected when plotting the frequency of the raw phenotype data from 2017 and 2018 (data not shown). Additionally, the values of the parents were distributed across almost the entire EDV span. A correlation analysis was conducted on the raw phenotypes (Table 2). The correlation coefficients ranged between 0.55 to 0.87. In general, the correlation coefficients between the disease phenotyping in 2017 and 2018 were high. An ANOVA revealed a significant difference in PM severity between the two different assessment years, 2017 and 2018. Furthermore, significant differences in PM severity were detected between all individuals in the four populations (data not shown).

**TABLE 2:** Correlation table of PM data for the entire population assessed across two years with two scorings (S) and two replications (R).

	**2017 1S_1R**	**2017 1S_2R**	**2017 2S_1R**	**2017 2S_2R**	**2018 1S_1R**	**2018 1S_2R**	**2018 2S_1R**	**2018 2S_2R**
2017 1S_1R	1							
2017 1S_2R	0.68	1						
2017 2S_1R	0.70	0.67	1					
2017 2S_2R	0.65	0.71	0.74	1				
2018 1S_1R	0.59	0.75	0.65	0.60	1			
2018 1S_2R	0.58	0.75	0.62	0.61	0.87	1		
2018 2S_1R	0.63	0.55	0.67	0.66	0.68	0.63	1	
2018 2S_2R	0.59	0.55	0.66	0.67	0.64	0.66	0.87	1

### Population Structure

**FIGURE 3:**
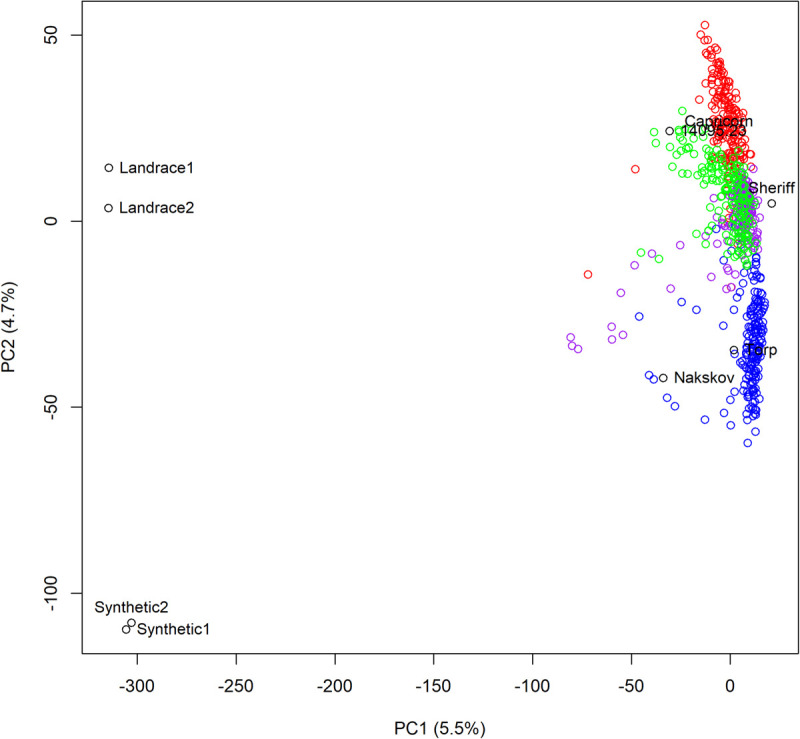
A principle component analysis of the four multi-parental populations. The first two principle coordinates are plotted against each other. The percentage variation explained for each coordinate is indicated in the axis titles. Population 1 is red, 2 is blue, 3 is purple, and 4 is green.

To investigate the population structure, a PCA was conducted on scaled and centered genotypes using all the lines from the multi-parental populations including the parents and the donor lines. The PCA revealed that the largest genetic difference in the data set was between the donors and the parental cultivars (Figure 3). The multi-parental populations were situated in between donor and parental cultivars, but much closer to the parental cultivars than to the donors. The second largest genetic difference in the data set was between the Chinese landraces and the synthetic lines – a difference that is reflected in the position of the multi-parental populations on the PC2. The multi-parental population scores on PC2 was in the same order as their respective donors scores on PC2 reflecting that the difference among the multi-parental populations was due to the different donors (Figure 3).

### GWAS

A genome-wide association analysis was performed to reveal loci on the wheat genome associated with PM resistance. In total, 8,902 mapped markers and 726 wheat lines were used in the GWAS. The distribution of the mapped markers across the wheat genomes revealed that the B genome contained most of the markers, whereas the D genome contained the least mapped markers. In total, 3481 markers were assigned to the A genome, 4221 to the B genome and 1,200 to the D genome.

**FIGURE 4:**
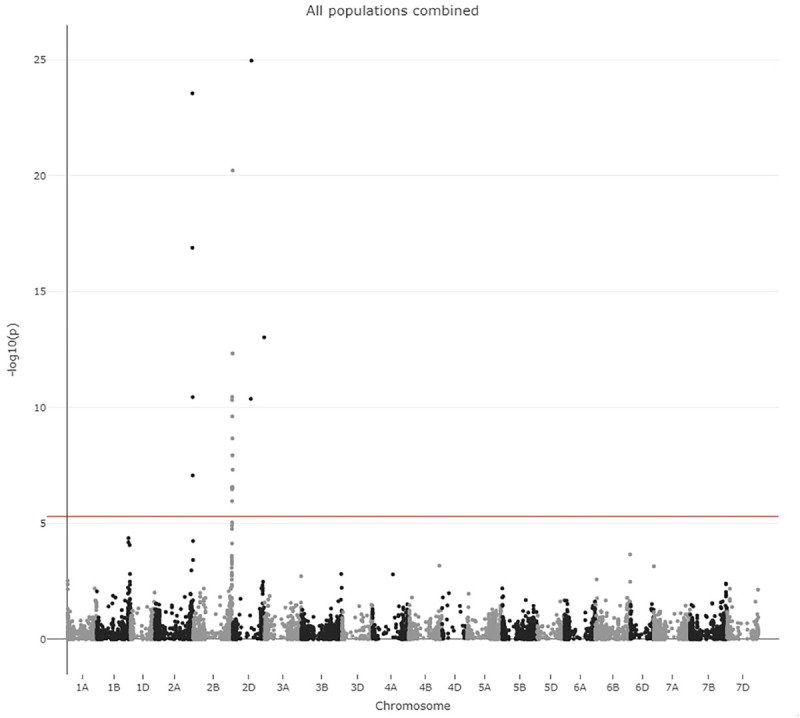
Manhattan plot from GWAS. The LOD score and chromosome number is indicated on the axis. The LOD score threshold of 5.3 is indicated by a red line.

Initially, the populations were analyzed separately for significant associations between marker genotypes and PM resistance. The output from the GWAS analysis performed on the separate populations revealed identical significant associations for all four populations (Supplementary Figure 1). Therefore, it was decided to perform GWAS on all populations together to obtain a more robust analysis. A Manhattan plot was generated to visualize the GWAS results and the QTL (Figure 4). In total, 22 SNP markers with LOD scores above the threshold 5.3 were detected (Supplementary Table 2), which indicates the markers being significantly associated to PM resistance. The results from the analysis of the alternative EDVs resulting from a weighted BLUP did not substantially differ from the presented analysis (results not shown).

All markers with a significant LOD score were analyzed by ANOVA to reveal whether the effect on PM resistance was significant. The 22 significantly associated markers were distributed across the three homoeologous chromosomes 2 from the A, B, and D genome (Figure 4). The three significant markers on 2D seemingly represented two different QTLs 381 Mpb apart. Thus, the markers that are significantly associated with PM resistance pointed to four different putative QTLs. The mapping information we used was derived from blasting the marker sequence to the wheat reference genome, and 11 of the 22 markers had more than one 100% hit representing at least two chromosomes (Supplementary Table 3). To investigate whether these four putative QTLs were segregating independently in our populations or represented one QTL on a single chromosome we investigated LD between the most significant SNP from each putative QTL both in the combined population (Table 3) and in each of the four populations (Supplementary Table 4). All combinations of the four markers displayed high LD ranging from 0.80 to 0.96 and probably represents a single QTL (Table 3).

**TABLE 3:** Linkage disequilibrium estimates between the top SNPs associated with the four putative QTLs when all the populations were combined.

	**SNP.2D-1**	**SNP.2B-1**	**SNP.2A-1**
SNP.2D-2	0.80	0.85	0.76
SNP.2D-1		0.91	0.96
SNP.2B-1			0.87
SNP.2A-1			

Single marker analyses were conducted on the most significant SNP marker (Table 4). The genotype of the marker is noted; a green cell indicates the positive allele, i.e., the allele resulting in the lowest disease score. The percentage explained of phenotypic variance of the QTL was 43%.

**TABLE 4:** Single marker analysis of the best marker.

**QTL**	**Marker name**	**Physical position (bp)**	**Haplotype**	**PM mean (EDV)**	**No. of lines**	***R*^2^**
Q.Pm.2D-1	SNP.2D-1	380924869	cmyk]0.47,0,0.88,0AA	−3.80	362	43%
			GG	5.14	304	

*Marker name, the physical position of the marker, genotype, where the green color indicates positive alleles, mean value of EDV based on PM scorings, number of lines with the noted genotype and the percentage explained of phenotypic variance (*R*^2^) are indicated. NA indicates that data is not available.*

The genotypes of the nine different parental and donor lines and the mean EDV for each line are shown in Table 5. The commercial cultivar “Sheriff” was the only line containing the positive allele in the most significant of the detected markers. The two synthetic lines possessed the lowest mean EDV.

**TABLE 5:** Overview of the genotypes of the parents and their mean EDV.

	**Q.Pm.2B-1**	**Mean EDV**
Synthetic I	**GG**	*rgb*].42,.753,.482−5.6493
Synthetic II	**GG**	*rgb*].388,.745,.482−5.9193
Chinese Landrace I	**GG**	*rgb*]1,.922,.518−0.7018
Chinese Landrace II	**GG**	*rgb*].973,.412,.4210.8655
Capricorn	**GG**	*rgb*].973,.412,.42−0.8185
Nakskov	**GG**	*rgb*].996,.784,.4942.4228
NOS 14095.23	**GG**	*rgb*].976,.427,.42410.5917
Torp	**GG**	*rgb*].98,.525,.4438.286
Sheriff	cmyk]0.8,0.02,0.96,0**AA**	*rgb*].541,.788,.49−4.5842

## Discussion

New sources of resistance against wheat diseases are needed to meet the demand for resistant wheat cultivars, i.e., to keep pace with the ongoing adaptation of plant pathogens to wheat when grown at large scale. Additionally, the need is equally high for genomic tools to incorporate newly identified resistance resources. Numerous studies have been conducted, searching for new resistance genes against PM (Bennett, 1984; Hovmøller, 1989; Hartl et al., 1999; Yi et al., 2008). However, no studies have used a multi-parental population for the task. In this study, four multi-parental populations were used for identifying and mapping new sources of resistance toward PM and for identifying both molecular markers closely linked to the resistance genes/QTL and the plant material that harbor these new sources of resistance. By using Chinese landraces and synthetic wheat lines as donors in the multi-parental populations, small proportions of alleles from these lines will be present in the final population. Theoretically, the population will consist of a genetic background from the parental cultivars and few alleles from the donor lines.

In general, multi-parental populations are believed to have little or no within population genetic structure and they are therefore highly suitable for great precision in fine mapping (Thépot et al., 2014). This is in agreement with what we observed in this study. As can be seen from Figure 3, very little genetic structure was detected within and between the four multi-parental populations. The low level of genetic structure among multi-parental populations with very different donors is not surprising as a high proportion of the genomes of the multi-parental population lines originated from the elite cultivars. The elite cultivars were related and lay close to each other in Figure 3. Therefore, the differences among the four multi-parental populations were small compared to the genetic differences among their donors. The lack of a strong population genetic structure makes the populations highly suitable for association studies, since populations with strong genetic structure also tend to give false positives (Ewens and Spielman, 2001; Dickson et al., 2010).

High correlations of PM assessment results between years and replications were observed (Table 2). Thus, the phenotypic data appeared to be highly suitable for association analysis. An ANOVA test revealed significant variations in disease severity between years and individuals. The disease data of 726 lines ranged from 0 to 68% PM severity in 2017 and from 0 to 50% in 2018. Differences in disease severity between year 2017 and 2018 might be due to environmental differences between different growing seasons. According to a national meteorological database provided by Aarhus University, records from a local weather station close to the field site at Jyndevad showed that 46 mm precipitation fell in May 2017 vs. 6 mm in 2018 and that the precipitation in June 2017 was 136 mm vs. 39 mm the following year. In addition, the relative humidity (RH) was 75% in May 2017 vs. 66% in 2018 and it increased in June 2017 to 80 vs. 39% in 2018. A difference in temperature was also recorded with an average high temperature of 17 and 19°C in May and June 2017 (highest recorded temperature was 24°C), respectively, whereas 21°C was measured in the corresponding months in 2018 and highest temperature records of 27°C in both months. Although the trial and surrounding sites were irrigated when needed, the large differences in precipitation, humidity and temperature between 2017 and 2018 is the likely cause of the difference in disease severity, although differences in the PM populations in the two years cannot be excluded. This would correspond well with PM development favored by high humidity >70%, cool and wet weather conditions (Wiese, 1978; Thomas et al., 2018). An earlier study reported a negative correlation between PM severity and humidity (Tang et al., 2017). This variation could also be due to changes in the pathogen population due to favorable conditions to some pathotypes. Significant variations between individual lines were confirmed by the frequency distribution of the EDVs for PM (Figure 2). The variance clearly shows that the multi-parental populations contain lines that are highly susceptible and lines that are highly resistant to PM and in all scoring dates and years we found high narrow sense heritability, making the populations ideal for association studies on PM resistance. The majority of the lines from population 2 and 3 were skewed toward the more negative EDVs. This indicates that major genes for PM resistance might be present in these multi-parental populations.

In this study, several loci on the homologues chromosome 2 revealed highly significant associations to PM resistance. A LOD score value above 20 was detected for one marker on each of chromosomes 2A, B, and D. Similar observations have been seen in previous QTL studies in wheat (Quarrie et al., 2006; Pushpendra et al., 2007; Huo et al., 2018). Thus, identification of homoeologous loci is not uncommon. Chromosome 2A, B, and D are homoeologous chromosomes, which are defined as chromosomes in related species that originated following allo-polyploidization (Glover et al., 2016). Thus, the chromosomes were completely homologous in the ancestral species. It is therefore quite likely that the causative gene for PM residence in our study is present in all the three homologous chromosomes. However, as the LD between these markers were at minimum 0.80 (Table 3), they probably represent a single QTL on one of the chromosomes. The amount of explained phenotypic variance was high, compared to similar association studies on PM (Li et al., 2014). Therefore, qualitative genes probably confer the resistance to PM. Earlier studies have identified 58 resistance genes to PM on all wheat chromosomes. Eight genes were previously mapped to chromosome 2B of which, four genes *Pm6*, *Pm33, MIZec1*, and *Pm5055* were mapped to 2BL (Jørgensen and Jensen, 1973; Mohler et al., 2005; Zhu et al., 2005; Saidou et al., 2016) and four genes, *Pm42*, and *Pm26, MlIW170*, and *MI5323* were mapped to 2BS (Rong et al., 2000; Hua et al., 2009; Liu et al., 2012; Piarulli et al., 2012). Additionally, a recent study identified resistance toward PM in German winter wheat cultivars on chromosome 2B at a position of 730 Mbp (Mohler and Stadlmeier, 2019). This QTL might be identical to the QTL that we identified. However, additional analyses are needed to confirm whether the QTLs in this study are allelic to previously reported resistance genes on chromosome 2B. In general, chromosome 2B appears to be a hotspot for disease resistance genes. Several resistance genes for yellow, leaf and stem rust have been identified and mapped to the long arm of chromosome 2B (Zhang et al., 2009; Cheng and Chen, 2010; Singh et al., 2011; Rouse et al., 2012; Li et al., 2014).

A transgressive segregation was observed, since more extreme phenotypes were observed in the segregating population compared to the parental and donor lines. This was further confirmed with the frequency distribution data, where several multi-parental lines were more resistant to PM than their parental and donor lines (Figure 2). In this study, the donor line Synthetic II was the most resistant line when comparing only the four donor lines. However, 12 lines from multi-parental population 3, where Synthetic II was the donor, were more resistant than this synthetic line. It is very likely that the PM resistance from the donor line in combination with the QTL we found in this study is the cause of this observation. This is based on the fact that the most resistant parental line, “Sheriff,” is ranked as number 75 out of 180 lines in total in population 3. Thus, 74 lines are more resistant than “Sheriff.” However, population 1 and 4, which contains the Chinese Landraces as donors, also show significantly associated QTL on chromosome 2A, B, and D even though the Chinese Landraces are not among the most significant resistant lines (Figure 2). Thus, this suggests that the QTL we detected most likely originates from “Sheriff,” which was included as the last crossing parent in all the four populations. Several QTL with low all frequency of the positive allele or small effects on the resistance may have contributed to the genotypes with the transgressivesegregation. Given the existence of minor unidentified QTL, genomic selection could be an option to identify lines with higher PM resistance taking into account the effect of all markers included in the multi-parental populations. Genomic selection analysis is beyond the scope of this study, but such analyses will be applied in future studies. Nevertheless, the chosen methods in this study successfully identified a QTL with large effects, which can be used for marker-assisted breeding.

## Conclusion

In this study we identified a QTL on one of the homologous wheat chromosomes 2A, B, and D in four multi-parental populations. The LOD scores of the associated markers, as well as the explained phenotypic variance were high compared to similar GWAS studies. We have shown that performing GWAS using multi-parental populations is highly valuable for identifying new sources of resistance against PM. Furthermore, the multi-parental populations provided not only knowledge about the resistance, but also about molecular tools and plant material that can be utilized in marker-assisted selection. Additionally, the four wheat multi-parental populations produced in this study are highly suitable for association studies of other traits than PM resistance.

## Data Availability Statement

The datasets presented in this study can be found in online repositories. The names of the repository/repositories and accession number(s) can be found below: Sarup, Pernille (2020): Phenotypes for powdery mildew GWAS winter wheat. figshare. Dataset. https://doi.org/10.6084/m9.figshare.12961811.v1. Sarup, Pernille (2020): Genotypes for powdery mildew GWAS in winter wheat. figshare. Dataset. https://doi.org/10.6084/m9.figshare.12961796.v1.

## Author Contributions

NN analyzed the data, prepared the figures, and wrote the manuscript. PS assisted in writing, analysing and preparing figures. TT designed the practical experiments and assisted in writing. TT and JR-A set up the practical experiments and collected the data by phenotyping. JA, MH, AJ, LJ, and JO planned the setup, formulated the theories, and supervised the project. All authors critically discussed the results and contributed to the final manuscript.

## Conflict of Interest

The study was performed in a collaboration between Aarhus University and the plant breeding company Nordic Seed A/S. NN, PS, JA, AJ, and JO were employed by company Nordic Seed A/S. The remaining authors declare that the research was conducted in the absence of any commercial or financial relationships that could be construed as a potential conflict of interest.

## Footnotes

1 https://www.upov.int/edocs/tgdocs/en/tg003.pdf
