# Supplementary figures and images for: Multi-Parental Populations Suitable for Identifying Sources of Resistance to Powdery Mildew in Winter Wheat
Source: Front Plant Sci. 2021 Jan 21;11:570863. doi: 10.3389/fpls.2020.570863 (PMC7859110; doi:10.3389/fpls.2020.570863)

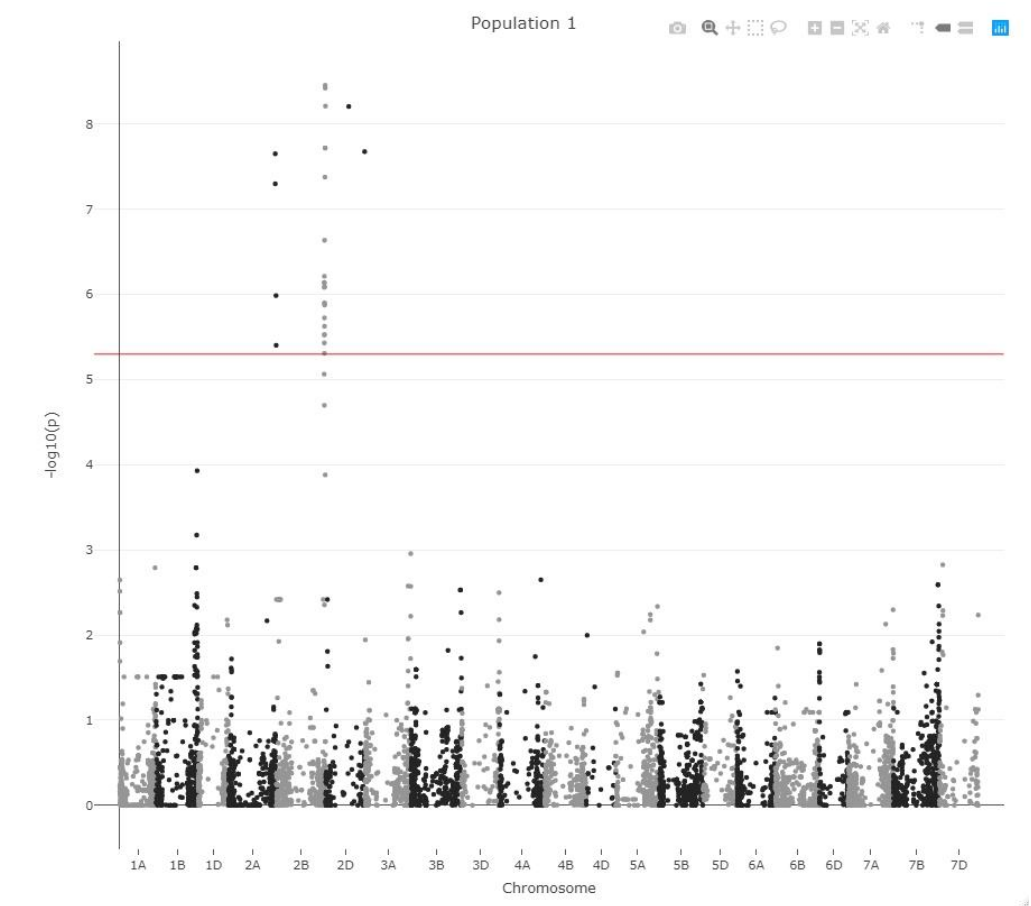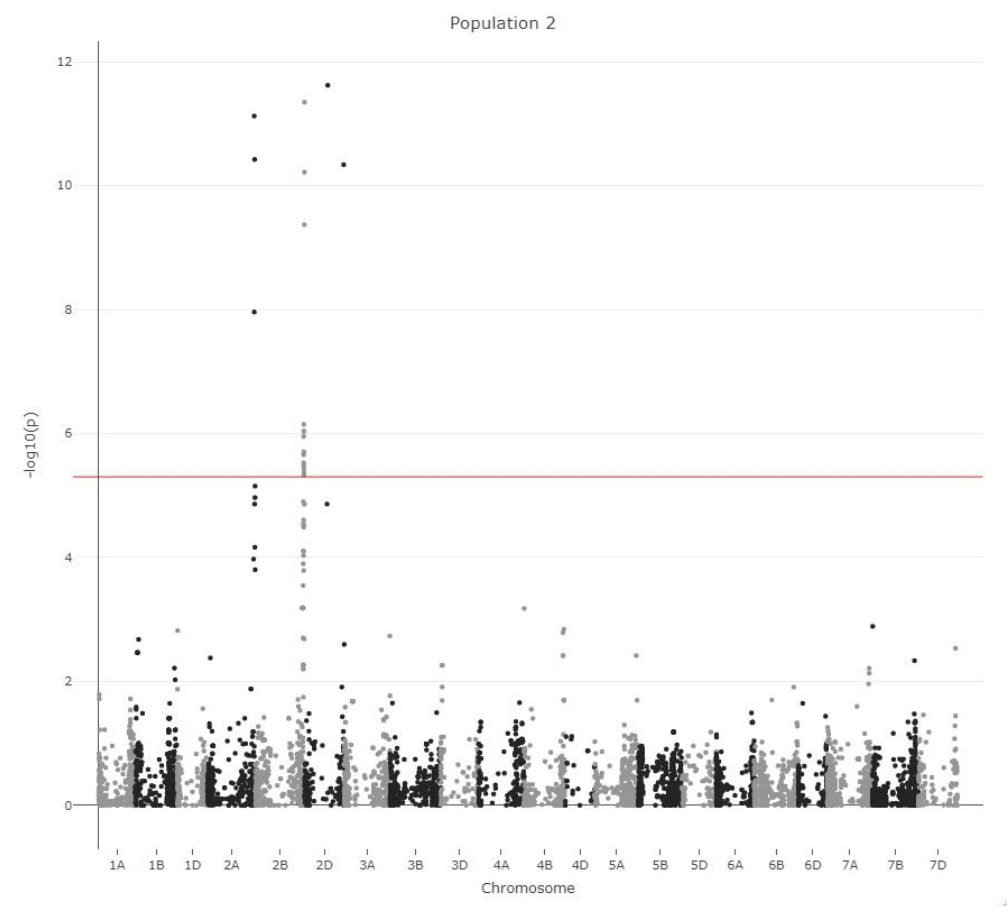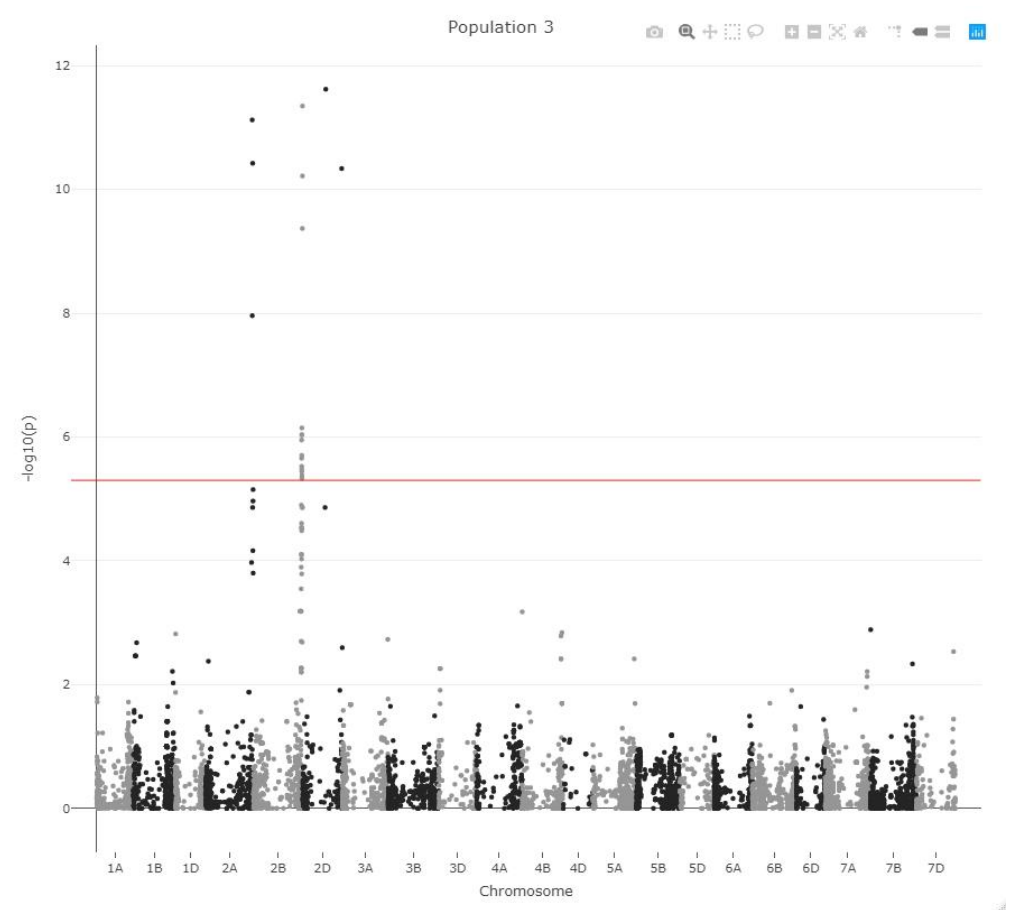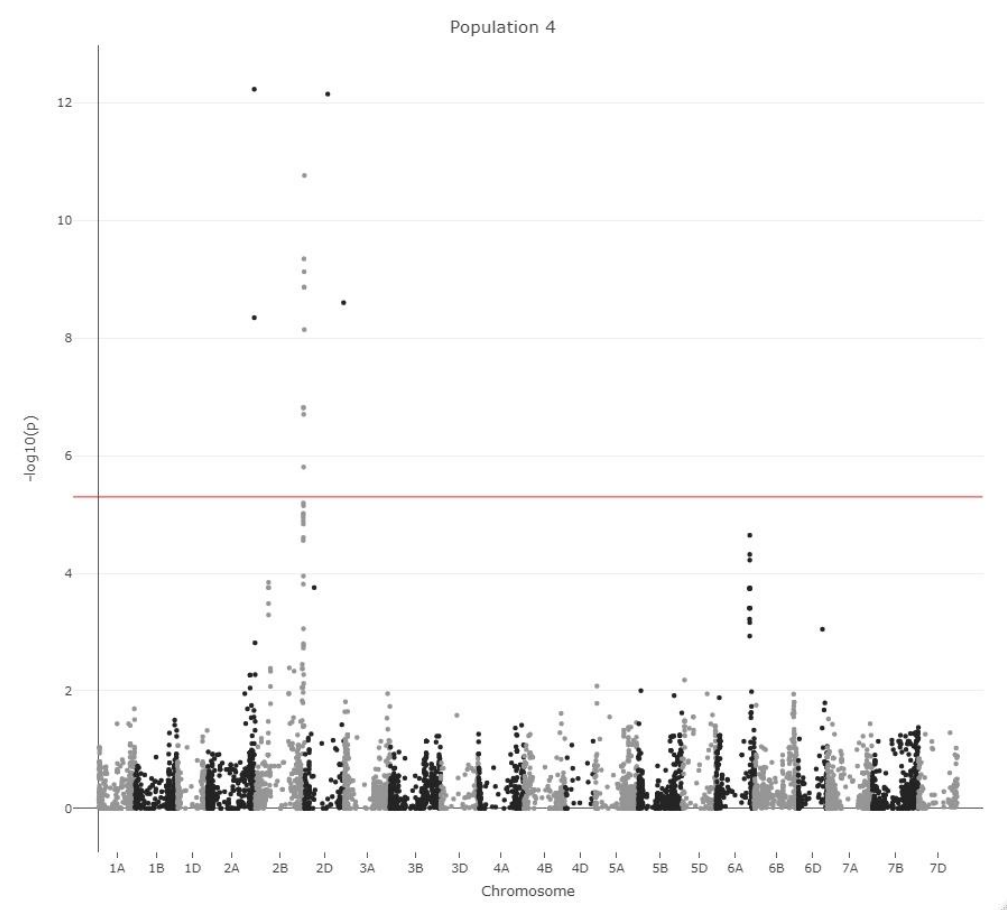

Supplement: Supplementary file 1 [file Data_Sheet_1.PDF]
