# Supplementary material for: Multi-Parental Populations Suitable for Identifying Sources of Resistance to Powdery Mildew in Winter Wheat
Source: Front Plant Sci. 2021 Jan 21;11:570863. doi: 10.3389/fpls.2020.570863 (PMC7859110; doi:10.3389/fpls.2020.570863)
Supplement: Supplementary file 2 [file Table_1.docx]

Supplementary table 1 Field and lab results of multi-parental population donor lines for powdery mildew, yellow rust and fusarium head blight.

| **NAM line donor** | **Source** | **Desirable traits** | **Pedigree** | **PM-09** | **YR-09** | **FHB-09** | **DON-09** | **NIV-09** | **Zea-09** |
| --- | --- | --- | --- | --- | --- | --- | --- | --- | --- |
| iKORN177 | Chinese Landraces | PM, YR | (((I-korn 177*Torp)*14095.23(sildig))*Capricorn)*Sheriff | 1 | 2 |  |  |  |  |
| iKORN113 | Synthetic line 1 | PM, YR, FHB | (((I-korn 113*Nakskov)*Capricorn)*Torp)*Sheriff | 0 | 0 | 0 | 271 | 2 | 2 |
| iKORN115 | Synthetic line 2 | PM, YR, FHB | (((I-korn 115*Torp)*Torp)*Capricorn)*Sheriff | 1 | 0 | 2 | 1786 | 177 | 21 |
| iKORN179 | Chinese Landraces | PM | (((I-korn 179*Nakskov)*Torp)*Capricorn)*Sheriff | 1 | 5 |  |  |  |  |

Powdery mildew (PM); yellow rust (YR); fusarium head blight (FHB); deoxynivalenol (DON), zearalenone (ZEA) and nivalenol (NIV)
