# Supplementary material for: Multi-Parental Populations Suitable for Identifying Sources of Resistance to Powdery Mildew in Winter Wheat
Source: Front Plant Sci. 2021 Jan 21;11:570863. doi: 10.3389/fpls.2020.570863 (PMC7859110; doi:10.3389/fpls.2020.570863)
Supplement: Supplementary file 5 [file Table_4.docx]

Supplementary table 4

Linkage disequilibrium (LD) between the most significant SNP from each putative QTL in each of the four populations.

LD calculated in population 1 (above the diagonal) and population 2 (below the diagonal)

|  | SNP.2D-2 | SNP.2D-1 | SNP.2B-1 | SNP.2A-1 |
| --- | --- | --- | --- | --- |
| SNP.2D-2 | - | 0.90 | 0.90 | 0.87 |
| SNP.2D-1 | 0.87 | - | 0.98 | 0.98 |
| SNP.2B-1 | 0.90 | 0.93 | - | 0.96 |
| SNP.2A-1 | 0.82 | 0.93 | 0.86 | - |

LD calculated in population 3 (above the diagonal) and population 4 (below the diagonal)

|  | SNP.2D-2 | SNP.2D-1 | SNP.2B-1 | SNP.2A-1 |
| --- | --- | --- | --- | --- |
| SNP.2D-2 | - | 0.72 | 0.76 | 0.72 |
| SNP.2D-1 | 0.70 | - | 0.91 | 0.99 |
| SNP.2B-1 | 0.84 | 0.82 | - | 0.90 |
| SNP.2A-1 | 0.64 | 0.93 | 0.76 | - |
